# Influenza Virus Infections in Polarized Cells

**DOI:** 10.3390/v14061307

**Published:** 2022-06-15

**Authors:** Beatriz Praena, Xiu-Feng Wan

**Affiliations:** 1 MU Center for Influenza and Emerging Infectious Diseases, University of Missouri, Columbia, MO 65211, USA; bpk9f@missouri.edu; 2 Department of Molecular Microbiology and Immunology, School of Medicine, University of Missouri, 1201 Rollins St., Columbia, MO 65211, USA; 3 Bond Life Sciences Center, University of Missouri, 1201 Rollins St., Columbia, MO 65211, USA; 4 Department of Electrical Engineering & Computer Science, College of Engineering, University of Missouri, 1201 Rollins St., Columbia, MO 65211, USA

**Keywords:** influenza A virus, polarized cell, sialic acid, N-glycan, O-glycan

## Abstract

In humans and other mammals, the respiratory tract is represented by a complex network of polarized epithelial cells, forming an apical surface facing the external environment and a basal surface attached to the basement layer. These cells are characterized by differential expression of proteins and glycans, which serve as receptors during influenza virus infection. Attachment between these host receptors and the viral surface glycoprotein hemagglutinin (HA) initiates the influenza virus life cycle. However, the virus receptor binding specificities may not be static. Sialylated N-glycans are the most well-characterized receptors but are not essential for the entry of influenza viruses, and other molecules, such as O-glycans and non-sialylated glycans, may be involved in virus-cell attachment. Furthermore, correct cell polarity and directional trafficking of molecules are essential for the orderly development of the system and affect successful influenza infection; on the other hand, influenza infection can also change cell polarity. Here we review recent advances in our understanding of influenza virus infection in the respiratory tract of humans and other mammals, particularly the attachment between the virus and the surface of the polar cells and the polarity variation of these cells due to virus infection.

## 1. Introduction

The influenza viruses in the Orthomyxoviridae family can be classified into the following seven genera: Alphainfluenzavirus (influenza A virus [IAV]), Betainfluenzavirus (influenza B virus [IBV]), Deltainfluenzavirus (influenza D virus [IDV]), Gammainfluenzavirus (influenza C virus [ICV]), Isavirus, Quaranjavirus, and Thogovirus [1], and these viruses are enveloped single-stranded and negative-sense RNA viruses. In addition to humans, IAV infects a variety of avian and other mammalian hosts (e.g., swine, canine, equine, and sea mammal species) [2]; IBV and ICV infect humans and swine [3,4]; IDV can infect domestic and feral swine, cattle, goats, sheep, camelids, buffalo, and equids [5,6,7,8,9,10,11].

IAV and IBV have eight genomic segments, coding for 10–18 proteins depending on the strain, including surface glycoproteins hemagglutinin (HA) and neuraminidase (NA), polymerase PB2, PB1, and PA, nonstructural proteins NS1 and NS2, and matrix proteins M1 and M2 [12]. IAVs are further separated into various subtypes based on the antigenic properties of HA and NA, and, to date, 18 HA (H1–H18) and 11 NA (N1–N11) have been identified [13,14]. Based on HA protein, contemporary IBVs are comprised of two lineages, B/Yamagata/16/88-like (Yamagata) and B/Victoria/2/87-like (Victoria), which have been co-circulating in humans since at least 1983 [15,16].

Seasonal human influenza (also known as the flu), mostly caused by IAV and IBV, is characterized by headache, muscular pain, cough, or fever symptoms. Most infected people generally recover within a few weeks, but occasionally, influenza can produce severe illness, possibly leading to death. It is estimated that, annually, there are from 3 to 5 million severe influenza infections worldwide [17], with from about 300,000 to 600,000 deaths [18,19]. Compared to IAV and IBV infection, ICV infection in humans is generally a mild upper respiratory disease, but it can be severe [20]. For children with ICV-associated, community-acquired pneumonia (CAP), clinical data were similar to those observed for children with IAV-associated CAP and worse than those observed for children with IBV-associated CAP [20]. Compared to IAV and IBV, ICV can also cause epidemics in humans but on a much smaller scale [21,22].

Cellular tropism plays an important role in human IAV infections. IAV triggers respiratory disease in humans and other mammals but may cause infections in both the respiratory and gastrointestinal tracts of avian species, particularly aquatic birds [23]. Human airway epithelial cells, including both ciliated and goblet cells, are susceptible to IAV infection with the expression of α2,6-linked sialic acids [24]. However, some recent studies suggest that sialylated N-glycans are not essential for the entry of influenza viruses. Compared to non-polarized cells, polarized epithelial cells have unique characteristics, such as the differential expression of IAV-specific receptors located on the apical and basal layers of these cells and polarized trafficking of cellular proteins [25,26], which play an important role in shaping virus infectivity in an in vivo system. This study is reviewing recent developments in influenza virus-cell receptor interactions, particularly with sialylated and non-sialylated glycans on polarized cells, and how these polarized cells respond to influenza virus infection. We will focus on studies on IAV infections in humans and other mammals.

## 2. Virus–Receptor Attachment and Viral Entry

### 2.1. Influenza Receptor-Binding Site

HA protein is composed of a trimer where each monomer is formed by a globular head domain (HA1) and a stalk domain (HA2). The receptor-binding sites of HA1 consist of 130-loop, 150-loop, 190-helix, and 220-loop [27]. The attachment between the viral HA receptor-binding site and the cellular glycan receptor initiates the virus infection cycle, and NA subsequently cleaves this receptor union and releases virions from infected cells. Previous studies show the importance of an equilibrium between the HA receptor-binding activities and the NA receptor-destroying activities, for which the sialidase function of NA leads to a hydrolytic cleavage of the glycosidic bond between neuraminic acid and sugar, allowing a rolling movement of the virion particle on the villi of epithelial cells [28,29,30].

### 2.2. The Respiratory Tract

**Figure 1:**
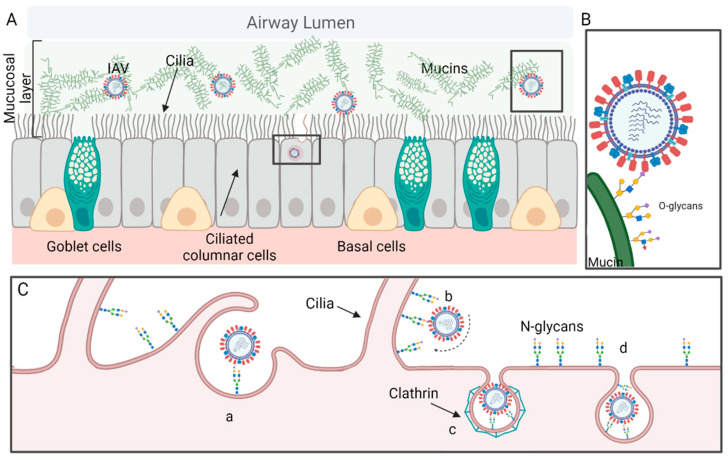
Infection of influenza A virus (IAV) in the human upper respiratory tract (URT). (**A**) heterogeneous polarized pseudostratified epithelium cells in human URT targeted by influenza viruses. (**B**) The first step of IAV infection. Hemagglutinin (HA) of influenza virion is attached to O-glycans present on the mucin surfaces. (**C**) After overcoming the mucus layer barrier, IAV reaches the apical surface of the ciliated epithelium cells. HA of the virions recognizes the glycan receptor present on the cellular membrane followed by viral entry mediated by endocytosis (a). An equilibrium of the receptor-binding activities for HA and the receptor-destroying activities of NA allows a rolling movement of the virion particle on the villi of the epithelial cell (b). Following cell surface attachment, the virus enters the cell through clathrin-mediated endocytosis (CME) (c) or clathrin and caveolae-independent endocytosis (d). The virions could exhibit either spherical or filamentous morphology.

The human airway can be divided into upper and lower respiratory tracts, both of which are susceptible to influenza virus infections, although the preference of virus replication between upper and lower respiratory tracts is virus subtype or even strain-dependent [31,32]. This respiratory system is lined with a gel layer composed mainly of water and glycoproteins, or mucins, secreted by goblet cells and submucosal glands [33]. To have a successful infection, the virus must go through the approximately 1–10 μm thick layer of mucus to reach the epithelium, which is constituted by a heterogeneous group of polarized cells, including ciliated columnar epithelial cells, basal cells, and secretory goblet cells (Figure 1A). Typically, only a small portion of inhaled pathogens can reach the epithelium because most of them are trapped by the mucus and subsequently brushed away by cilia through respiratory tracts into the pharynx for removal [34].

### 2.3. Attachment and Viral Entry

The influenza virus infection can be initiated by direct or indirect contact between influenza viruses and mucosal cells of the respiratory tracts, which are composed of epithelial cells and the underlying lamina propria. By direct contact infection, the virus can reach the mucosal cells by aerosols or droplets. The expiratory particles are typically categorized into aerosols (≤5 µm, suspended in air) or airborne droplets (>5 µm, and usually in liquid form), and aerosols are further separated and classified into fine aerosols (<1 µm) and coarse aerosols (1–5 µm). Airborne droplets travel 3–6 feet and typically infect the proximal mucosal surfaces of the upper respiratory tracts (e.g., mouth, conjunctiva, nasal mucosa, or other mucosal surfaces of the upper respiratory tract) [35,36] whereas aerosols can remain suspended in the air longer and disperse farther, and can lead to infections in both upper and lower respiratory tracts [37,38,39]. In addition, the influenza virus could also be spread by indirect transmission by contact with contaminated fomites [40,41].

To infect the epithelial cells, the virus needs to overcome mucosal barriers, which are laid above the epithelial cells and are composed of ~150 mucin-type O-linked glycoproteins (O-glycoproteins) [42]. The first virus binding in the respiratory tract typically takes place between virus HA and the O-linked glycans (O-glycans) on the mucins on the surface of the mucosa [43] (Figure 1B). Then an asymmetric distribution of HA and NA on the surface, both in the virions with spherical morphology and, more evidently, in those with a filamentous morphology, allows efficient binding cleaving by NA and facilitates the virus rolling on microvilli penetrating the mucus layer (Figure 1C) [44].

After reaching and binding to the receptor on the epithelial cells, the virus enters the cell through clathrin-mediated endocytosis, clathrin and caveolae-independent endocytosis [45] (Figure 1C), or macropinocytosis [46]. Endocytosis-dependent protein pathways have been extensively studied, but most in homogeneous monolayers of cells and a limited number in either heterologous polarized cell populations or in vivo.

As soon as the virus particle enters the coated vesicles, the virus is transferred to the endosome where it will encounter acidic pH conditions. As the endosome matures and acidifies, protons enter the virion through M2 channels and create an acidic interior environment [47], which leads to a conformational change in the viral HA and, consequently, a fusion between the viral envelope and the endosome surface and the expelling of the viral genome into the cytoplasm [48,49]. The pH conditions that trigger the fusion with the endosome are around a pH value of 5, varying slightly among the different influenza virus strains. The stability of HA in the acidic pH environment exhibited by polarized respiratory epithelium could be an advantage for influenza infection against mucus, the concentration of ions and salts, and even the pH itself. It is considered that the upper respiratory tract of mammals is a slightly acidic environment; therefore, the virions with a lower pH of HA activation can produce a more successful infection. This hypothesis has been tested in cells of the primary epithelium of the human respiratory tract where viruses with a less stable HA in low pH undergo an early activation outside the cells resulting in a failed infection [50]. In another study, swine IAVs isolated from 2009 to 2016 showed the following different acidic activation traits: gamma-clade viruses had less stable HA proteins (activation pH 5.5–5.9) than pandemic clade (pH 5.0–5.5) [51]. Both in vitro and in vivo studies suggested that a relatively stable HA protein (pH 5.5–5.6) was necessary for efficient replication and airborne transmission for subtype H1N1 swine IAV in humans.

## 3. Receptor of IAVs

**Figure 2:**
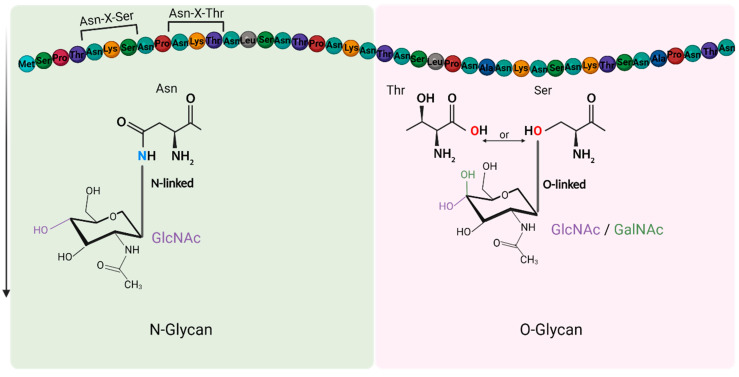
Illustration of N- and O-glycans associated with IAV binding. N-glycan binds to a protein through the nitrogen of an amide in Asparagine (Asn) and the first carbon of N-acetylglucosamine (GlcNAc) (**left**). The N-glycosylation sites are located in the “marker sequence” Asn-XaaThr/Ser without a Proline in Xaa position. The O-linkage between a glycan and a protein form between the oxygen of a Threonine (Thr) or Serine (Ser) with the first carbon of N-acetylglucosamine (GlcNAc) or N-acetylgalactosamine (GalNAc) (**right**).

The oligosaccharide chain can be attached to a protein through an O-linkage with the oxygen of Serine (Ser) or Threonine (Thr) (as an O-glycoprotein) or through an N-linkage with the amide of Asparagine (Asn) (as an N-glycoprotein). The sites of N-linked glycosylation (N-glycoprotein) depend on the amino acid sequence surrounding Asn, being the “marker sequence” Asn-Xaa-Thr/Ser [52], with the Xaa position occupied by any amino acid other than proline, and such a marker sequence is also known as sequon (Figure 2). Sialylated N-glycans are the most well-characterized receptors for influenza viruses and have been identified in the epithelia cells across the respiratory tracts of humans and other mammals [53]. On the other hand, the O-glycans are widely expressed in the mucins with an O-linked GalNAc, followed by other glycan residues such as galactose, GlcNAc, fucose, and sialic acid (Sia) [54]. The O-glycans, particularly those sialylated glycans on the mucins, can bind and trap the IAV, preventing the viruses from entering the epithelia cells (Figure 1B) [43]. Thus, it is important to study influenza virus-cell receptor interactions in polarized epithelial cells since only these polarized cells can produce mucus on their apical surface [34].

Although all four known influenza viruses (IAV, IBV, ICV, and IDV) are known to use Sias as receptors, the species of Sias are genera-dependent [55,56,57]. In this review, we will only focus on the receptors for IAVs.

### 3.1. Sialylated N-Glycans Receptors

IAV attachment initiates when the conserved HA receptor-binding site attaches to the terminal Sia of the host cellular sialylated N-glycan receptors. The monosaccharide Sia is a derivative of neuraminic acid with a nine-carbon structure and an N-acetyl group at C5, which could be an N-acetylneuraminic acid (Neu5Ac) or an N-glycolylneuraminic acid (Neu5Gc) [58] at the end of a glycan chain. For the majority of influenza glycan receptors, Sia is α-linked at C2 to galactose of a cellular glycoprotein or glycolipid. The α-linkage is well known to be associated with the host, tissue, and cell tropism of IAV infections, and human IAVs typically prefer an α2,6-linked Sia (galactose C6) and avian IAVs to an α2,3-linked (galactose C3) Sia [59,60].

Only a few studies have been conducted in which Neu5Gc, a common form of Sia, acts as an influenza virus receptor [61,62,63,64,65,66], while most studies have focused on Neu5Ac. Glycan microarray analyses suggested that avian, swine, canine, and equine IAVs have the ability to bind both Neu5Ac and Neu5Gc, whereas human IAVs typically do not bind Neu5Gc [67]. In human respiratory tracts, the epithelial cells do not express Neu5Gc, whereas those in swine, equine, and canine respiratory tracts do [61,65,68].

However, the virus receptor-binding specificities may not be static and continue to evolve during host adaptation. For example, the receptor-binding specificities of human seasonal H3N2 viruses have changed in the past few years, and glycan microarray analyses suggested that recent H3N2 viruses prefer long polylactosamine chains terminating in sialic acids, such as those α2,6-sialosides on extended LacNAc moieties, whereas those earlier ones prefer short, branched sialylated glycans [69,70,71]. Another study also suggested that recent H3N2 viruses exhibit increased recognition of complex sialylated N-glycans and non-sialylated phosphorylated high-mannose glycans [72]. The changes in glycan binding specificities in sialylated glycans are likely due to substitutions in the receptor-binding sites of virus HA caused by antigenic drift [69,71,73].

### 3.2. Other Glycan Receptors

**Figure 3:**
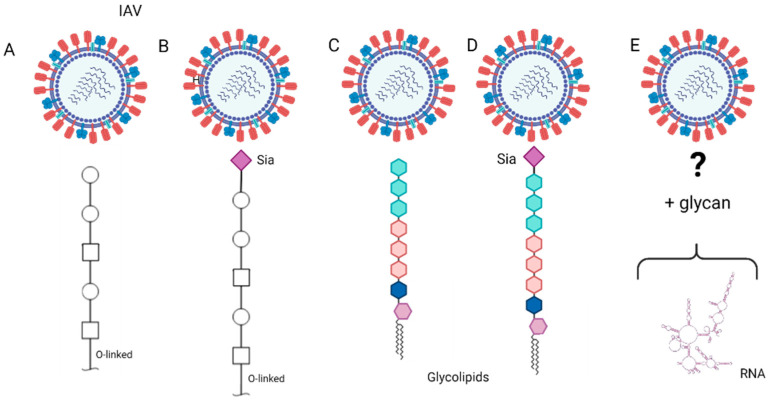
Illustration of possible binding interactions between influenza A virus (IAV) and those glyco-molecules other than N-glycans on the cellular surface in the human respiratory tract. The glyco-molecules can be O-glycans without sialic acid (Sia) (**A**), O-glycans with Sia (**B**), glycolipid without Sia (**C**), glycolipid with Sia (**D**), and glyco-RNA (**E**). Among these glyco-molecules other than N-glycans, the interactions between O-glycans and IAV are more documented in literature but those for glycolipid and glyco-RNA are still unknown [81].

Although they are well documented to be the receptors for IAV, sialylated N-glycans are not essential for influenza virus entry into the host cells [74]. In addition to N-glycans, it is plausible that influenza viruses may enter the cells through attachment to O-glycans (non-sialylated or sialylated) [75,76] (Figure 3A,B), glycol-lipids (non-sialylated or sialylated) (Figure 3C,D), and/or even non-glycan receptors (Figure 3E).

In the desialylated MDCK cells, in which all sialic acids in glycoproteins and glycolipids were depleted by exogenous neuraminidase (sialidase) treatment, the infectivity of multiple strains of H1 and H3 viruses remained evident [74]. The data from this study support that there may be a non-Sia molecule that plays a role as a secondary receptor to induce the endocytosis pathway during virus entry. Another study carried out in a mutant CHO cell line suggested that N-linked glycoproteins but not Sias expression alone are important for efficient infection, although Sias expression can enable virus binding. In this study, the Lec1 cells, a mutant CHO cell line that is deficient in terminal N-linked glycosylation but with an expression of Sias on the cell surface, influenza viruses showed initiation of binding and fusion but did not cause efficient infection [77]. Thus, despite its key role in virus attachment, Sia (e.g., those in O-glycoproteins or O-glycolipids) alone may not be sufficient to ensure a successful virus infection. These data suggest that non-N-linked molecules, sialylated or not (e.g., O-linked glycoprotein), could instead be intervening in a precursor attachment step as the first binding receptor. In addition, these data demonstrate that N-glycoprotein expression is required for efficient influenza virus infection.

Similar results were obtained from various heterogeneous cellular systems. Subtype H5 influenza virus infection remains efficient in fully differentiated and polarized, normal human bronchial epithelial (NHBE) cells, which are treated with neuraminidase to be Sia α2,3 or α2,6 moieties depleted on the cell surface [78]. Of note, the epithelial cells in the human respiratory tract did not express simple Sias α2,3Gal on the cell surface but expressed α2,3Galβ1,3/4GlcNac Sias and other non-sialylated motifs [79]. Nevertheless, these data from NHBE cells further support the possibility of non-sialylated motifs as influenza virus receptors or the recognition of Sias with linkages other than α2,3 and α2,6.

A microarray with glycans released directly from human lung tissue in a matrix showed that H3N2 viruses between 2001 and 2017 presented binding avidities to high-mannose phosphorylated glycans or non-sialylated poly-N-acetyllactosamine, which could have a functionally distinct role from N- or O-sialoglycans [73]. This study suggested shotgun microarrays, which are printed with cellular glycans (instead of synthetic glycans), as a powerful tool to characterize receptors in the target cell or tissue samples.

Recent studies suggest that, in addition to N-glycoprotein, O-glycoprotein, glycolipids, and other molecules on the cellular membrane or in the cells can be sialylated. For example, voltage-gated Ca^2+^ channels can be sialylated, and these sialylated channels are not only recognized by the influenza virus but are also involved in viral infection [80]. Another study showed small noncoding RNAs carry sialylated glycans and are present on the cell surface [81], although the role of glycoRNA glycosylation in virus attachment and infection is unclear. These results suggest it is possible that there are alternative sialylated receptors for the known N-glycoproteins.

In summary, although Sia has been known as an influenza virus receptor for decades, these recent studies indicate that the molecular recognition and virus entry for influenza viruses is not fully elucidated. It seems clear that viral-cell attachment and virus entry, both important steps for the virus infection cycle, can take place as two isolated events. Nevertheless, to fully understand the biological role of glycan receptors, studies shall be designed to integrate virus-cell attachment with viral entry.

## 4. Influenza Virus Infection Can Change the Complexity and Polarity of Cells

Cell polarity refers to spatial asymmetry in cell morphology and/or organization of cellular components. The monolayer of epithelial cells in the mammalian respiratory tract is well known for its apical-basal polarity, which is characterized by the asymmetrical distribution of cellular components and functions between an apical surface facing the external environment and a basal surface attached to the basement layer. The epithelial cells are typically perpendicular to the basal membrane, and the mitotic cells are also primarily perpendicular but with a small portion parallel to the basal membrane during cell division [82]. Enabled by the polarized actin and microtubule cytoskeleton, the perpendicular structure of the epithelial cells is organized with an apical-basal axis, a polarized trafficking machinery, and an intercellular tight junction (TJ) [26]. The polar cells possess TJs in a basolateral localization where the membranes of two adjacent cells merge to form a barrier. Appropriate functioning of this polar complex will be essential for correct molecular trafficking and communication between adjacent cells [25,83]. Viruses such as influenza can take advantage of this polar complex and use it for their own purposes of entry into the cell.

At least three protein complexes are reported in maintaining cell polarity, including the CRUMBS/PALS1/PATJ complex, the PAR3/PAR6/aPKC complex, and the SCRIBBLE/DLG/LGL [25], of which the SCRIBBLE/DLG/LGL complex also plays a role in preserving the proper function of the apical junctions, such as the TJ in mammalian epithelial cells. Appropriate functioning of this polar complex is essential for correct molecular trafficking and communication between adjacent cells [25,83]. In addition, correct cell polarity and directional trafficking of molecules are essential for the orderly development of the system, and damage or loss of either of these two properties can lead to cellular malfunction.

In alveolar epithelial cells, human H1N1 and avian H5N1 viruses would be able to infect both through the apical and basolateral surfaces of the epithelium. However, human H1N1 infections were more efficient when the virus was inoculated from the apical than from the basolateral side, and opposite results were shown in avian H5N1 infections, causing more efficient infections than H1N1 when the virus was inoculated from the basolateral side, but no difference was observed when virus inoculation was performed from the apical side. These results indicate potential transmission of H5 viruses through routes such as viremia in addition to aerosol or aerosol droplets, as well as the likely dissemination of viruses through the basolateral side to the blood [84]. It is plausible that virus replication in the polarized epithelial cells could weaken the TJ and cause cell death, facilitating viruses to access the basolateral aspect of microvascular endothelial cells.

**Figure 4:**
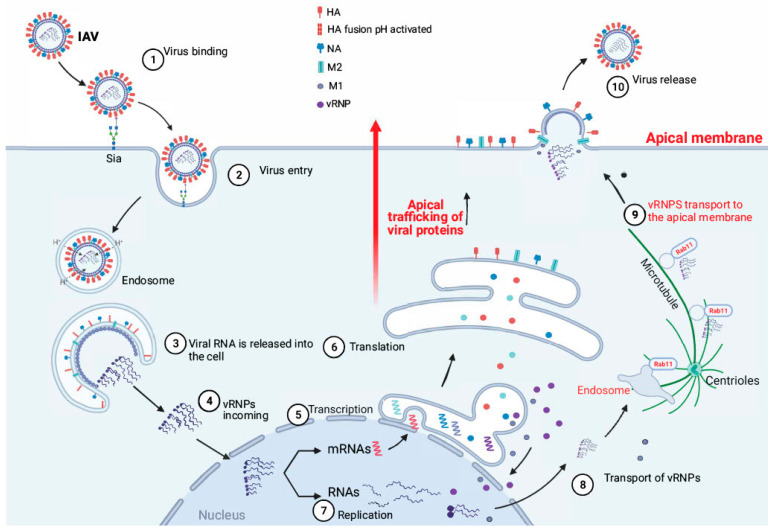
Apical transport during influenza A virus infection. The viral infection cycle starts with a recognition of a glycan receptor (e.g., sialylated glycan) on the cellular membrane by viral HA. The virus enters the cell through endocytosis (Figure 1); in the acidic environment in the endosome, protons traverse the viral membrane through M2, facilitating viral fusion and release of the viral genome into the cellular cytoplasm. Then the viral ribonucleoproteins (vRNPs) are transported into the nucleus followed by transcription and replication. Next vRNPs are cotrafficked with Rab proteins and use Rab11-dependent vesicular and microtubule-based transport pathway to be transported from the nucleus to the plasma membrane through the pericentriolar recycling endosome. The membrane proteins HA, NA, and M2 proteins traffic from the Golgi to the apical membrane. Finally, the viral particle buds and is released by the action of NA to the extracellular media.

The association between cell polarity control and virus infection can be seen in the review by Thomas and Banks [85]. Microbial infection can destabilize and disrupt cell polarity and may even induce oncogenesis [86]. However, all IAV proteins show a polar localization pattern, as follows: HA, NA, and M2 are localized on the apical plasma membrane in polarized epithelial cells, and M1 and vRNP are transported to the apical plasma membrane along with the other proteins for an effective virion assembly (Figure 4). The polar movement of the viral proteins through the cells is related to the free vesicular-dependent movement of cellular macromolecules [87,88]. Influenza viral RNPs are cotrafficked with Rab proteins and use Rab11-dependent vesicular and microtubule-based transport pathways to transport from the nucleus to the plasma membrane (Figure 4) through the pericentriolar recycling endosome [89]. Thus, it is important to maintain cell polarity for an infective influenza virus infection. A recent study showed that M2 protein apical membrane targeting is essential for effective transmission, and M2 protein basolateral plasma membrane (M2-Baso) or endoplasmic reticulum (M2-ER) significantly reduced the number of viable virions to be generated, as shown more in primary human nasal epithelial cells than in MDCK cells [90]. During acute respiratory distress syndrome caused by IAV, viral infection can cause Na/KATPase, which is typically localized to the basolateral membrane, to be re-distributed to the apical surface due to potential interaction with viral M2 protein [91]. Relocation of Na/KATPase outside the basolateral membrane can lead to a reduction or even loss of the ability to maintain sodium gradients and consequently cause excessive fluid and pathogenesis in the lung.

On the other hand, for an effective virus infection, it also seems important for these cells infected by the influenza virus to maintain polarization and structural integrity. In MDCK cells with Lgl2 protein overexpression, which destroys cell polarity by impairing the barrier function of TJ, the influenza virus cannot have smooth traffic of the viral ribonucleoproteins (vRNPs) to export NP viral protein out of the nucleus, suggesting the impact of cell polarity on virus replication [92]. This observation is in line with previous studies showing that subtype H5N1 avian influenza viruses manipulate cell polarity to inhibit pro-apoptotic functions through an interaction of a conserved 4-amino-acid-residue PDZ-ligand-binding motif (PBM) (at the C-terminus of the viral NS1) to proteins SCRIBBLE and DLG [85,93]. The viral NS1 is recolocalized with SCRIBBLE and DLG proteins within perinuclear puncta, and this was shown to be associated with the mislocalization of Lin7C from the plasma membrane to the cytoplasm. The Lin7C forms a tripartite complex with Dlg1 and MPP7 in the CRUMBS complex, which is known to be involved in cell polarity, and mislocationzation of Lin7C caused an impediment of the apoptotic function, and the cell-cell junction was disrupted. The MAGI proteins are found at TJ and are associated with the maintenance of adherents and TJ, and, in addition to Scribble and DLJ, NS1 can bind to MGAI-1, MAGI-2, and MAGI-3 through PBM [85,93,94]. On the other hand, it was demonstrated that there is direct binding of the NS1 and the polar protein MAGI-1 that perturbs interferon-β signalization, thereby contributing to immune evasion and virus release [95]. Of note, the NS1 binding efficiency to SCRIBBLE, DLG, and MGAI proteins is strain-dependent and was observed only in the isolates with amino acid consensus motif “ESEV and RSKV” but not those with RSKV, KSEV, or EPEV in the PDM sequences [85,93].

## 5. Importance of Studying Influenza Virus Infection in Polarized Cells

In summary, influenza viruses manipulate cell polarity while taking advantage of cell polarity for polar transport of viral proteins to achieve efficient virus assembly, as has also been demonstrated in many other viral infections [85].

Compared with the in vivo models, in vitro cell cultures are more frequently utilized in studying influenza virus infection due to their wider accessibility and lower costs. The development of in vitro human cell models allows for representative studies without invasive testing in humans (or other mammals). For example, H1N1 infection can be detected by measuring the volatile metabolite produced by primary human tracheobronchial epithelial cells [96], and the similar environment between primary polarized tracheobronchial cells and the cells in vivo provides the ability to extrapolate the results [97]. Compared with the homogeneous established cell lines, the heterogeneous polarized cells better represent the apically polarized cells of the human and other mammalian airway systems. The common heterogeneous polarized cells are either immortal or primary epithelial cells collected from nasal, tracheal, or lung tissues in humans or other mammals (e.g., swine and ferret). The example of established and polarized cell lines commonly used in influenza studies include those from bronchi (e.g., Calu-3), immortalized/transformed primary epithelial cells (e.g., 16HBE14o-), or cells from alveoli (e.g., A549) grown on porous filter support in the ALI system [98]. These polarized cells have been invaluable resources in assessing influenza virus infectivity, understanding the natural history of influenza viruses, unveiling molecular mechanisms underlying influenza cell and tissue tropisms, and characterizing physiological dynamics associated with virus pathogenesis during virus infection [96,97]. Table 1 summarizes variations in the expression of Sias, the primary IAV receptors, across commonly used cells in influenza research and vaccine development. Overall, compared to those immortal cells, primary cells, which are heterogeneous and polarized, would have the most similar glycan receptor expression patterns to in vivo systems and ex vivo tissues, for example, showing differential expression of α-2,6 Sia and α-2,3 Sia on their apical and basolateral surfaces or differential expression depending on the cell type in the heterogeneous system.

**Table 1:** Expression of α-2,6 Sia and α-2,3 Sia on different cell cultures. The expression of α-2,6 Sia and α-2,6 Sia was detected by fluorescence microscopy via lectin binding. +++: Highly detected. ++: Detected. +: Low detected. +/−: Rarely detected. -: Not present.

Culture *	Host	Polarization	Cell Type	α-2,6 Sia	α-2,3 Sia	References
HAE	Human	Y	Ciliated	++	++	[99,100]
			Nonciliated	+++	+	
hTEC	Human	Y	Ciliated	++	+++	[24]
			Nonciliated	+++	-	
HNTEC 3D	Human	Y	-	+++	++	[101]
			
Ex vivo	Human	Y	Ciliated	++	+++	[24,100]
			Nonciliated	+++	-	
			Basal	-	++	
PBEC	Swine	Y	Ciliated	+	+	[102,103]
			Nonciliated	+++	-	
			Basal	+	+++	
NE	Swine	Y	Ciliated	++	-	[103]
TE	Swine	Y	Unknown	++	-	
LE	Swine	Y	Unknown	++	+/−	
FTE	Ferret	Y	Ciliated	+++	-	
			Nonciliated	+	+++	[104]
PD	Human	N	-	+++	+	[99]
HNTEC 2D	Human	N	-	++	++	[101]
MDCK	Canine	N	-	+	++	[105]
MDCK-SIAT1	Canine	N	-	+++	+	[106,107]
MDCK-London	Canine	N	-	++	+	[107]
hMDCK	Canine	N	-	+++	-	[108]
16HB14o-		N	-	++	+++	[109]

* Human airway tracheobronchial epithelium grown in ALI system (HAE), human tracheoepithelial cells (hTEc), primary human normal tracheal epithelial cells (HNTEC), lung and tracheobronquial ex vivo tissue (Ex vivo), primary porcine bronchial epithelial cells grown in ALI system (PBEC), explant tissues from nasal (NE), tracheal (TE), bronchial (BE), and lung (LE) tissues, ferret trachea epithelial cells grown in ALI system (FTE), poorly differentiated cells from HAE (PD), Madin-Darby canine kidney cell line (MDCK) and human bronchial epithelial cell line (16HB14o-).

Although the established cell lines are easier to manipulate than the heterogeneous polarized primary cells, some immortal cell lines exhibit incomplete differentiation or polarization, leading to the absence of TJs or no mucin formation [83]. It is not trivial to develop and maintain those primary heterogeneous polarized cell cultures, most of which require a viable and easier-to-handle culture in an air-liquid interface (ALI) to allow the cells to be sufficiently differentiated. Other common challenges include short cell half-life, non-homogeneity between cellular cultures from different patients or donors [110,111], and/or undesired glycan receptor distributions at the apical and basal surfaces [100,101,102].

The distribution of influenza virus-associated glycan receptors such as α2,3-Sia and α2,6-Sia is shown to be different between the less-differentiated HNTEC in 2D culture and the well-differentiated HNTEC in ALI 3D cultures. The 2D HNTEC exhibits a higher expression of α2,3-Sia—avian influenza receptors—compared to α2,6-Sia, whereas, after cell differentiation in the 3D culture, the cells express higher levels of α2,6-Sias, particularly located in the basal and apical layers. Thus, the results indicated that well-differentiated HNTEC-derived 3D cultures expressed more human influenza virus-like receptors similar to those in tracheal tissue [101]. As a result, compared to the primary normal human tracheal epithelial cells (HNTEC) cultured in 2D, the well-differentiated HNTEC in ALI 3D cultures are more susceptible to H1N1 infection.

Another study showed that non-ciliated human pseudostratified mucociliary airway epithelium (HAE) cells present a higher proportion of α2,6-Sia expression in the apical layer, whereas the ciliated HAE cells in ALI express both α2,6-Sia and α2,3-Sia at similar levels. Avian IAVs can exclusively infect ciliated cells, whereas human IAVs show an unequal infection. An H3N2 virus from the 1968 pandemic period can infect ciliated cells, but recent H3N2 seasonal viruses preferentially infect non-ciliated cells [100].

In a porcine differentiated epithelial system, α2,6-Sia is expressed on the apical surface of differentiated epithelial cells, and the basal cells mainly express α2,3-Sia, and such a distribution is comparable with that in a human differentiated epithelial system [102]. However, undifferentiated cells showed increased expression of α2,3-Sia on the apical surface that involutes with differentiation.

Overall, the influenza virus infection studies performed in these polar epithelium cells help illustrate how influenza virus tropism evolves, adaptation over time, and a more representative picture of real influenza infection in humans. However, some studies suggest that the expression of mRNAs or activation of different essential molecules for the correct behavior and development of the in vivo system may not be reproducible in established cell lines [112,113]. As an alternative, explant tissues are used in studying influenza cell and tissue tropisms [23,65,114,115,116] and drug development [96].

In summary, all these studies highlight the importance of studying influenza virus infection in a heterogeneous cellular system, which would ideally reveal accurately both biological and physiological properties of the heterogeneous populations of polarized cells in the airway of humans and other mammals [117].

## 6. Conclusions and Perspectives

In humans and other mammals, the respiratory tract is represented by a complex network of polarized epithelial cells, forming an apical surface facing the external environment and a basal surface attached to the basement layer. These cells are characterized by differentially expressed proteins and glycans that serve as receptors during influenza virus infection. Attachment between these host receptors and the viral surface glycoprotein HA initiates the influenza virus life cycle, and NA cleaves the receptor and releases virions from infected cells. To date, the most studied influenza receptors are the sialylated N-glycans, but in addition to them, other molecules, such as O-glycans and non-sialylated glycans, may be involved in virus-cell attachment, and their role in virus infections needs to be elucidated.

The study of viral attachment in a polar and heterogeneous cell system provides representative data of host infection. In addition to receptor binding, where differential localization of virus receptor expression is observed in these cells, correct cell polarity and directional trafficking of molecules are essential for the orderly development of the system and affect successful influenza infection, and influenza infection would also change cell polarity. Thus, precise characterization of the influenza virus infection cycle using heterologous polarized cell systems is important and can improve our understanding of the disease and aid in the development of effective countermeasures against virus infection.

## Data Availability

Not applicable.
